# Attitudes and Consumer Behavior toward Foods Offered in Staff Canteens

**DOI:** 10.3390/ijerph17176239

**Published:** 2020-08-27

**Authors:** Ewa Czarniecka-Skubina, Hanna Górska-Warsewicz, Joanna Trafiałek

**Affiliations:** 1 Department of Food Gastronomy and Food Hygiene, Institute of Human Nutrition Sciences, Warsaw University of Life Sciences (WULS), str. Nowoursynowska 166, 02-787 Warsaw, Poland; joanna_trafialek@sggw.edu.pl; 2 Department of Food Market and Consumer Research, Institute of Human Nutrition Sciences, Warsaw University of Life Sciences (WULS), str. Nowoursynowska 166, 02-787 Warsaw, Poland; hanna_gorska_warsewicz@sggw.pl

**Keywords:** staff canteens, food quality, pro-healthy menu, customer service, customer behavior

## Abstract

The aim of our study was to analyze the attitudes of employees toward food offered in staff canteens, to analyze their eating behavior and the factors determining the choice of meals in staff canteens. The survey was conducted on a sample of 600 adult respondents, who patronize staff canteens in Warsaw, Poland. The research enabled a detailed and comprehensive assessment of consumer behavior toward the use of staff canteens, as well as their opinions on the functioning of the canteens and meals offered. Factors determining the frequency of use of canteens, type of meals, and factors influencing the use of such establishments were identified. Respondents assessed the quality and variety of meals, with the size of portion being the most valued. They were satisfied with the functioning of the canteens and had very few comments. However, they pointed out the need for an increase in the variety of meals, including the availability of vegetarian and vegan dishes. Our research enabled us to establish consumer profiles based on the reasons for not using staff canteens and comments about staff canteens. Patrons of staff canteens were referred to as “canteen enthusiasts” or “canteen medium-enthusiasts”, who are “snack lovers”, as well as “food choice-oriented” or “quality-oriented”. The results provide the basis for practical implications for owners or managers of staff canteens, part of whose work it is to analyze the needs and expectations of their potential customers.

## 1. Introduction

The staff canteen is defined as a place where food is served in a company or enterprise so that employees can eat during their working hours [1]. They could be part of institutional and commercial food services, and they are located in government facilities, enterprises, industry plants, healthcare facilities (hospitals, nursing homes), prisons, schools and child care organizations, military settings (canteens and rations), meals on wheels, and the workplace of canteen staff [2,3].

The increased importance of workplace nutrition is attributed to the fact that workplaces are one of the key channels of interventions to reduce the incidence of chronic diseases in adult populations and improve public health via direct and indirect measures [4,5,6,7,8,9]. It is also important to take a break in the context of emotional exhaustion, job satisfaction, and organizational behavior [10]. Staff canteens are crucial because they could offer the intake of healthy, well-balanced food according to modern guidelines [11,12]. This is also related to the development of good eating habits [11,13], which also depend on acceptability, food neophobia, food intake, culture, and gender [14,15].

As previously mentioned, staff canteens are a good place to shape eating behavior and develop proper attitudes toward healthy nutrition. A study conducted among full-time employed females suggested that having lunch at the staff canteen improved their eating habits, and meals served at the staff canteen may serve as a model for an optimal diet [11]. At the same time, providing healthy food and drink at the workplace can improve calorie intake at work. Employee eating activities in staff canteens can be a part of health promotion programs, including the promotion of physical activity in nutrition programs, involving employees in nutritional planning, and adapting meals according to the needs of employees [16].

Previous studies were mainly related to the restaurant and suggested that food quality (its presentation, healthy options, taste, freshness, the temperature of meals [17,18,19,20,21,22], physical environment (e.g., design, music, lighting)) and service (e.g., professional skills, staff reliability, and interaction with customers) are mainly components of overall restaurant service and quality [17,18,19,20].

Our article fills a research gap in the literature by evaluating the quality of staff canteen nutrition, employee eating behavior, their preferences, and factors determining their choice. Previous research focused mainly on the factors influencing the choice of restaurants and other catering establishments, as well as school or university canteens. In general, the factors influencing consumer decisions about eating out are related to food (taste, cost, accessibility of food) and quality of services [23,24], not only to nutritional value [24]. Other authors stated that factors such as marketing and promotion, as well as price, are not so important [25,26,27,28]. According to the research, the type of catering establishment could influence consumer hierarchy. For example, the experience in ethnic restaurants is more important than the choice of food and as important as its quality [29], while hygiene is more important than food [30,31,32]. In the previous decade, introducing a healthy menu in catering establishments and taking diet into account were important factors in the decision-making process when choosing a dining place [23,26,33,34]. Jang et al. [35] observed an important segment of consumers and called them “health-conscious consumers”. There are very few publications [4,8,16,32,36,37,38,39,40] referring to staff canteens, especially canteens located in office buildings.

Therefore, the aim of our research was to analyze the attitudes of employees toward food offered in staff canteens, as well as to analyze their eating behavior and the factors determining the choice of meals in staff canteens. The following research questions were formulated:

- What are the motives (factors) of employees choosing staff canteens?
- What consumer profiles can be identified according to the frequency of using staff canteens?
- How do employees evaluate the canteens in terms of equipment, service, and menu attractiveness?

## 2. Materials and Methods

### 2.1. Characteristics of Staff Canteens

The research was conducted in five staff canteens in Warsaw, which were located in office buildings. They were similar in terms of the size of consumer rooms, the number of places for consumers (75–100), and the number of meals served daily (200–300).

The number of operating staff present in the consumer room was related to the lunchtime and ranged from 2–4 people. The canteens were open from 9:00 a.m. to 4:00 p.m. Most people participated in customer service between 12:00 and 2:00 p.m. Dietary dishes (one set each) were offered by only two restaurants. The daily menu of each restaurant offered two soups, from 3–5 meat dishes, based on various types of meat (e.g., pork, beef, poultry), in various forms: main courses, special dishes, chef’s dishes, and fish and vegetarian dishes. In addition, there were days when oven-baked dishes, grilled dishes, pasta, and pancakes were prepared in front of customers, as well as weeks with national cuisine from around the world.

### 2.2. Sample Size Determination

Our research was conducted using a sample of 600 adults from canteens in office buildings in Warsaw, Poland, which was managed by an international catering operator. In 2018, there were 2653 catering establishments in Warsaw, of which there were 408 canteens, located mainly in schools and universities [41]. The international big catering operator on whose canteens the study was conducted serves 16 staff canteens in Warsaw.

Sample size (*n*) was determined using a single population proportion formula [42], based on the following assumptions:

$$n=\frac{{\left(z_{a/2}\right)}^{2}\;\times \;p\left(1-p\right)}{d^{2}},$$

involving the proportion of the number (31.3%; *p* = 0.313) of studied staff canteens on the basis of all canteens served this catering company 31.3% (*p* = 0.313), among 408 canteens in Warsaw [41], standard normal distribution confidence interval (*z_a_*_/2_) (1.96), and margin of error (*d*) = 0.05. Taking 5% as the response, the minimal total sample size became 330. We achieved 600 completed questionnaires, which exceeds the minimum number of participants.

### 2.3. Data Collection

We designed the questionnaire on the basis of available questionnaires [43,44] and our previous research related to foodservice [45,46,47], taking into account the specificity of staff canteens. The questionnaire was assessed by determining its repeatability. The reliability of the questionnaire was validated using its internal consistency. We estimated it using Cronbach’s alpha coefficient. In our case, Cronbach’s alpha value was 0.75, indicating adequate internal consistency [48,49].

A pretest of the questionnaire through a pilot study (*n* = 15) was performed within the population of interest. This group was not added to the main research. All problems were identified, and the questionnaire was completed and corrected. The data were collected by the authors using the PAPI (pen-and-paper interview) method. Inclusion criteria of respondents were as follows:

1. Each respondent who agreed to participate in the survey was invited to complete the questionnaire. If necessary, explanations were provided.
2. Adults over 18 years of age, regularly using the full offer of the canteen, not suffering from diseases requiring a special menu offer.

The exclusion criterion of respondents was people who were in the canteen for the first time. Only those respondents who met the recruitment criteria participated in the study.

The participants in the research were a convenient sample of consumers. Research was carried out during the person’s stay in the staff canteen. The respondents were free to participate in the questionnaire. Based on the fact that the research is non-invasive and the details of the participants remain undisclosed, this research does not fall within the remit of the Helsinki Declaration.

The questionnaire consisted of two parts (Table S1). The first part of the questionnaire included 12 questions relating to consumer behavior in the staff canteens and consumer attitudes toward food offered in the canteen. Consumer behavior was analyzed based on the frequency of canteen visits, factors influencing the use of canteens, time the canteen was used, time usually spent eating in the canteen, frequency of dish and beverage consumption, assessment of material factors in the canteen, evaluation of customer service, and recommendation of the canteen to other people. Three questions were connected with the menu: evaluation of menu, consumer attitudes to implementation of the pro-healthy menu, and reasons for the rare use of canteens. The second part of the questionnaire was related to respondent sociodemographic details: gender, age, education, workplace, assessment of financial status, and length of work in the current place.

### 2.4. Characteristics of Respondents

The characteristics of the respondents are presented in Table 1. The study covered young female and men with higher education, working mainly as office employees or middle management staff. They declared different financial statuses; however, no one considered their financial status to be “bad”. Most of the respondents (72%) worked for less than two years or 2–5 years in the office building where the rated staff canteens were located. The respondents were free to participate in the research.

### 2.5. Data Analysis

The statistical analysis of the results was performed using Statistica software (version 13.3 PL; StatSoft Inc., Krakow, Poland). The ANOVA test and multi-dimensional cluster analysis were used. Significance of differences between the values was determined at a significance level of *p* < 0.05.

Two multi-dimensional cluster analysis calculations were performed: hierarchical cluster analysis and principal component analysis (PCA). The goal of our cluster analysis calculation was to build a tree diagram where the answers given by participants were most similar in the specific cluster. We used the cluster analysis to determine consumer profiles based on the reasons for not using staff canteens and their opinions about restaurants. In the cluster analysis of consumer profiles, some of the reasons given for not using staff canteens included fast food preferences, skipping a meal, other restaurant preferences, eating home-made food, food delivery, eating at home, snacking, and cafeteria preferences. In the cluster analysis of consumer opinion profiles, features such as price, service, hygiene, promotion, organization, and quality were taken into account. PCA was used to identify sociodemographic factors (principal components) affecting the staff’s requirements of food. We established the number of main components based on the scree plot (scree test). As a part of data pre-treatment, correlation eigenvalues were calculated. Then, the eigenvectors of the correlation matrix were calculated. The values and signs of eigenvectors provided information about the direction strength of the correlation between individual component variables. On the other hand, a five-point scale was applied for 10 items in the assessment of material factors in canteens, for eight items in the evaluation of customer service, and for 21 items in menu assessment.

## 3. Results

### 3.1. Consumer Habits in the Range of Use of Staff Canteen Services

The respondents used the services of staff canteens with varying frequencies. Gender, age, financial status, and workplace did not influence the frequency of using canteen services (*p* > 0.05).

Depending on this frequency, three consumer profiles were created:

- Canteen enthusiast—consumers very often (4–5 times a week) used canteens, with higher education (49.7%, *p* = 0.004);
- Canteen medium-enthusiast—respondents used the staff canteens three times a week and belonged to various sociodemographic groups (17.8%, *p* = 0.004);
- Occasional canteen consumers—consumers used canteen services twice a week, once a week, or less than once a week, having secondary education (16%, *p* = 0.004);
- Unspecified/random canteen customers.

Respondents used staff canteens at lunchtime, but at different times of serving. Lunchtime in Poland is between 12:00 and 3:00 p.m. This was usually at the beginning (31.2%) or at the middle of lunch break (33.3%). The other customers (17.3%) usually came to the canteen at the end or had no fixed time for a meal (18.2%). The choice of time to use the canteen depended on the gender (*p* = 0.0003) and age of the respondents (*p* = 0.0486). Women and people aged 31–50 were more likely to use canteens at the beginning of lunchtime or had no fixed time, while men were more likely to come inside at the end of this time. People under 30 years used the canteen at half the lunchtime period.

Respondents usually spent 15–30 min (71.33%) or 15 min (18.83%) in staff canteens. Few people spent more time on a meal: 31–45 min (8.3%), 46–60 min (1.54%). The time spent on a meal in a staff canteen depended on the age of customers (*p* = 0.0313). Other sociodemographic data did not influence the time spent in a canteen (*p* > 0.05).

The typical components for lunch in Poland are soup and a main course including meat, a portion of carbohydrates, and salad. In staff canteens, the respondents most often bought vegetarian dishes (*n* = 554) with the highest frequency (three times a week) and main courses (*n* = 381) at a lower frequency (once or less than once), as shown in Table 2. The choice and frequency of eating particular dishes in the staff canteen depended on various demographic factors (Table 2). The choice of hot and cold breakfasts, salads, and fruit was significantly dependent on the financial situation of respondents. The purchase of soups and main courses was significantly dependent on the level of education and financial status of respondents. Purchase of vegetarian dishes depended on the age and education of the respondents. Statistically, people over 31 years of age and with higher education bought vegetarian dishes more often. Sandwiches were bought significantly more often by women, people with very good or good financial status, and people with higher education. Fast food dishes were significantly more often bought by people under 30 years of age and people with good financial status. The financial situation, age, and workplace significantly influenced the purchase of cakes/pies in staff canteens. Significantly more often, such a choice was made by office employees, people under 30 years of age, and people with good and average financial status. Purchases of cold and hot beverages depended mainly on the workplace of respondents. Often times, they were chosen by office employees and middle management. Purchases of desserts were not dependent on demographic factors.

### 3.2. Factors Affecting the Use of Canteen Services by Respondents

The respondents used the services of staff canteens mainly due to their location close to the workplace (76.5%), price (33.3%), and quality of services (33.2%). A smaller percentage of respondents indicated habits (19.7%), meetings (7.33%), and possibility of eating in a pleasant atmosphere (3%). Among other factors (13.5%, *n* = 81), respondents mentioned no other alternative and time to look for other places to eat a meal (*n* = 24), discounts bought by employers, possibility to get coupons, discounts (*n* = 11) for regular customers, and professional training in which they attended in the building where the canteen is located (*n* = 18). Moreover, they indicated the quality of dishes and services, the speed of service (*n* = 17), the freedom of use of the canteen, and a large selection of dishes, including the possibility of creating their own sets (*n* = 9), as well as the possibility of different portion sizes (*n* = 2).

**Figure 1:**
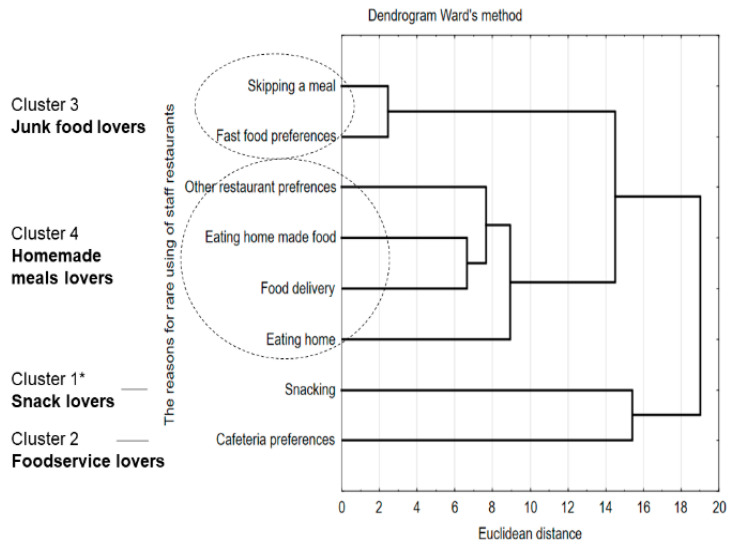
The analysis of consumer profile with the reasons for not using the staff canteens and the division into groups of reasons; * clusters were numbered according to the frequency of indications.

As a reason for the rare use of staff canteens, the respondents mentioned various factors. Cluster analysis made it possible to understand the reasons why employees gave up meals in the staff canteens. These are characterized into four profiles of such consumers, grouped into four clusters (Figure 1). The most numerous group of consumers was “snack lovers” who like ready-to-eat snacks and eat them between meals (cluster 1, 32.83%). The next group was “foodservice lovers”, who prefer other types of catering establishments, e.g., cafes (cluster 2, 27.83%). The third group was “junk food lovers”, made up of consumers with “incorrect eating habits”, e.g., skipping meals and who prefer food like fast food (cluster 3, 10.03%). The fourth and smallest group was “homemade meal lovers”. They were consumers preferring home food (consumed at home or brought to work) or food delivered from other places to home (cluster 4, 5.89%).

**Figure 2:**
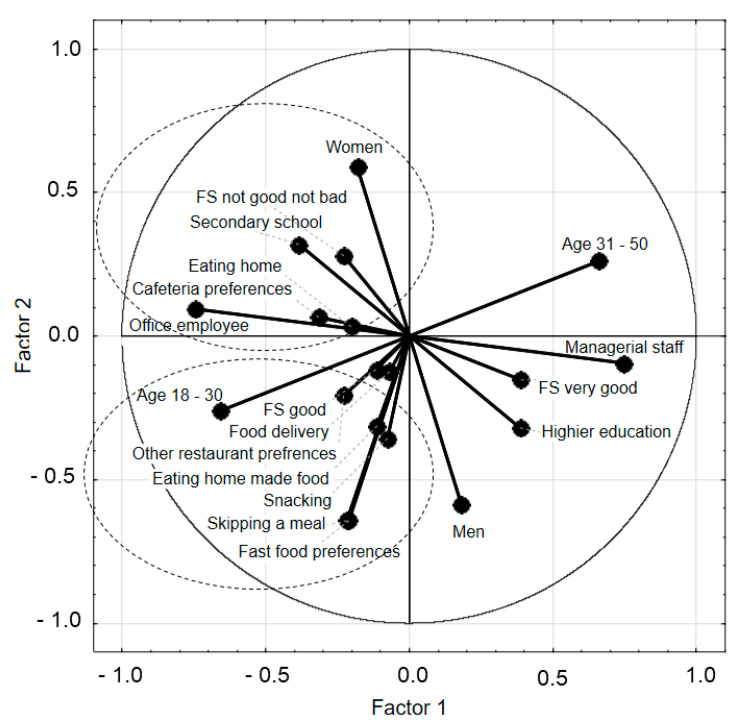
The analysis of consumer profile with the reasons for not using the staff canteens and the link of the indicated reasons and sociodemographic factors; FS—financial status.

An attempt was made to link the indicated reasons for the rare use of staff canteens by respondents with the sociodemographic factors of the respondents using the PCA analysis (Figure 2). Not all factors were related to the specific reasons given for avoidance of staff canteens. Only two groups of such factors were identified. The first group was young respondents aged 18–30 years old and those with good financial status, while the second group was women, people with secondary education, people working in offices, and people declaring the average financial situation. Respondents from the first group avoided the use of staff canteens for a variety of reasons. They preferred food in fast-food joints and restaurants (6% of the total), ordered delivery of meals (3.5%), or brought food from home (5.2%). They did not rule out skipping lunch either. In contrast, women and office employees, with an average financial situation, did not use staff restaurants because they preferred food at home (8.8%) or food in a cafe or a fast-food bar (6%).

Among very important elements of the functioning of the canteen, the respondents mentioned large portions and the quality of meals as important ones, including variety and refinement of meals. Among other elements, they also mentioned opportunity to relax, quality of service, low price of a meal, possibility of conducting promotional campaigns, discounts, the possibility of ordering a meal to the office or take-out, and traditional and special menus. Unimportant features of the service listed were the possibility of a unique composition of dishes and international cuisine menu (Table 3).

**Figure 3:**
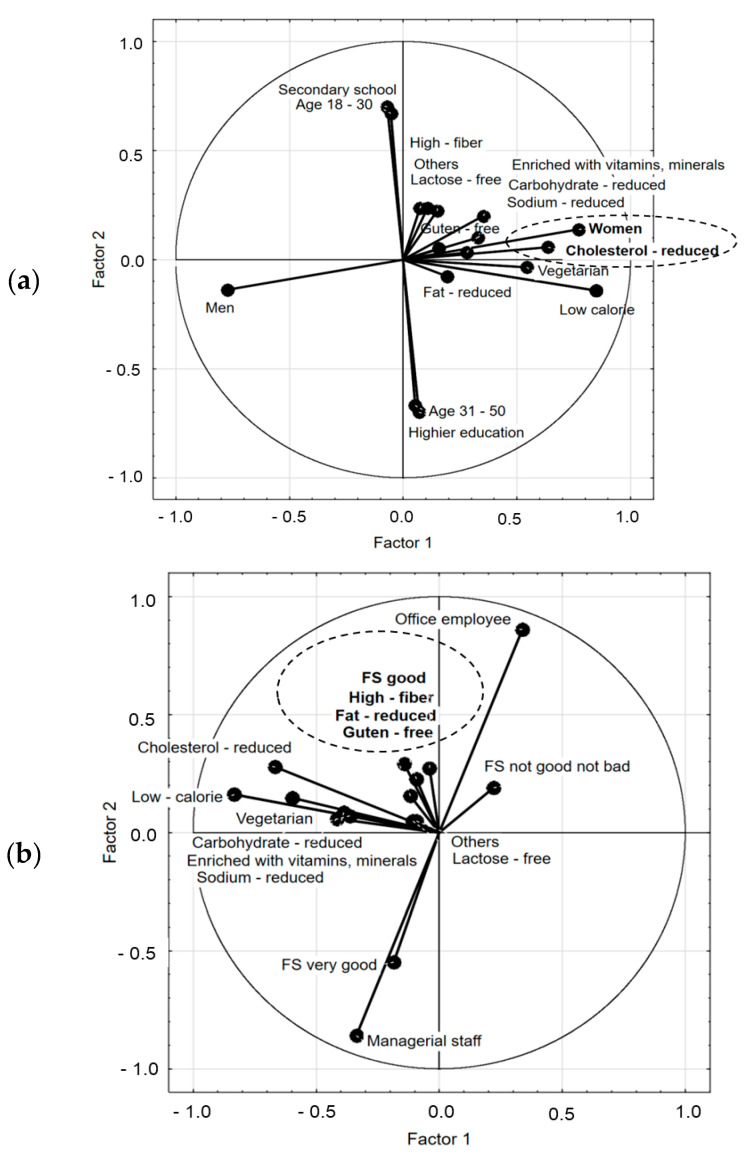
(**a**,**b**) The relationship between the consumer expectation toward the staff canteen meals and the consumer sociodemographic factors: (**a**) gender, age, education; (**b**) position and financial status of consumers; FS—financial status.

When asked about the importance of a pro-healthy menu in staff canteens, the respondents indicated a large number of different types of these dishes: low-calorie dishes (22.8%), vegetarian meals (13.0%), fat-reduced meals (12.17%), cholesterol-reduced meals (30.8%), sodium-reduced meals (21.2%), simple carbohydrate-reduced meals (15.2%), meals enriched with vitamins and minerals (15.7%), high-fiber meals (24.8%), gluten-free meals (16.7%), lactose-free meals (1%), and others (0.5%). PCA calculations indicated sociodemographic factors differentiating the requirements for meals in staff canteens (Figure 3).

Only women (Figure 3a) and individuals with good financial status (Figure 3b) had specific requirements concerning the offer of meals in staff canteen. Women wanted meals with low cholesterol levels. However, men had no requirements or preferences for meals in the staff canteens. People with both very good and average financial status had no specific preferences. High-fiber, reduced-fat, and gluten-free meals were preferred by many respondents. Factors that did not differentiate specific quality expectations into meals were identified. These were age, education level, and workplace. PCA calculations showed that those consumers who chose less salt also expected vitamin-enriched meals, as well as meals with reduced simple sugars.

### 3.3. Evaluation of Quality of Staff Canteens by Respondents

Respondents rated the quality of services in staff canteens which they use most often. Material factors, customer service, and menu were evaluated (Table 4).

Most material and customer service factors were rated as good (median 4). The location of the canteen was rated as very good (median 5). The features of staff canteen menu such as the attractiveness of the offer and availability of the menu, as well as taste, temperature, the appearance of dishes and size of portions, quality and prices of soups and main course, variety of meals, breakfast offer, meals prepared using the pro-healthy cuisine method, possibility of receiving various discounts, and ordering additional services were rated as good (median 4). Salad offer, variety of meals in the daily menu, opportunity to buy commercial products such as yogurt, prices in relation to the quality offered, readability and visibility of prices in the menu card, and information about discounts and promotions were rated as average (median 3). The lowest rates were achieved for the option of ordering take-out dishes (median 2).

In summary, 63.5% of the respondents were satisfied with the staff canteen services. The opinion of other respondents about services was different. About 25.3% of consumers were partially satisfied (answer: “neither yes nor no”), but 11.2% of respondents were rather not satisfied (“no” or “definitely no”) with the services. Recommendations for canteens were similar, whereby 55.3% would recommend canteens to others and 28.8% were hesitant, but 15.8% of respondents did not recommend canteens.

**Figure 4:**
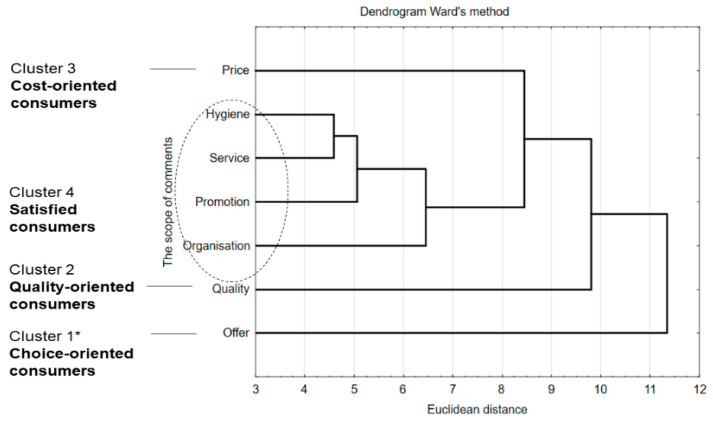
Consumer profile referring to comments about staff canteens; * clusters numbered depending on their importance for consumers.

Multidimensional calculations (cluster analysis) were used to analyze respondents’ opinions on staff canteens (Figure 4). This led to the definition of consumer quality requirement profiles. However, the calculations showed that employees using staff canteens had very few comments on the restaurant’s operations. The first and second consumer profiles concerned the quality of offered meals. They were “choice-oriented consumers” (profile 1) and “quality-oriented consumers” (profile 2) (cluster 1, 13%, and cluster 2, 9.83%, respectively). The third profile was “cost-oriented consumers”, expecting favorable prices for meals (cluster 3, 8%), and the fourth was “satisfied consumers”, who pay little attention to hygiene, service, and promotion (cluster 4, 2.06%).

It should be emphasized that canteen consumers focused on the health-promoting features of the food offered. They postulated the idea of increasing the variety of meals, including vegetarian and vegan dishes, salads, cooked vegetables, egg dishes, fish and poultry dishes, fresh fruits, and fresh fruit juices, as well as reducing the number of meat and flour meals, in favor of health-promoting light and low-fat dishes. Customers suggested reducing the number of dishes fried in favor of baked and cooked dishes, including steamed and grilled ones. The respondents pointed out that some elements of dishes are not tasty or too salty, or that they even have an unnatural taste, which suggests preparation from concentrates. According to the respondents, the use of additional substances was a cause of malaise and stomach problems after eating meals in staff canteens.

## 4. Discussion

### 4.1. Consumer Attitudes and Behavior in Staff Canteen

The interest in catering services, especially staff canteens, in Poland is increasing due to insufficient time, returning home late, and too many professional duties, as well as the increased income of the Poles [50].

The conducted research enabled a detailed and comprehensive assessment of consumer habits concerning the use of staff canteens, as well as their opinions on the operations of those establishments and meals offered. Factors determining the frequency of use of staff canteens, type of meals consumed, and factors influencing the use of such canteens were identified. Above 50% of respondents, who we called “canteen enthusiasts” or “canteen medium-enthusiasts” used the staff canteens regularly, every day or a minimum of three times a week. Other people used the canteen occasionally, whom we called “occasional canteen consumers”. Employees used staff canteens primarily for lunch. The use of this type of restaurant was primarily influenced by the workplace (76.5%), price (33.3%), and quality of services (33.2%). The four profiles of consumers who used staff canteens, in view of food choices, were identified as “snack lovers”, “foodservice lovers”, “junk food lovers”, and “homemade meals lovers”.

The behavior and habits of Polish consumers in staff canteens presented in our research are similar to those in other European countries, especially in large cities characterized by large populations. Over 50% of respondents used staff canteens regularly, every day or a minimum of three times a week. This is similar to the study in Helsinki, Finland (48–54%) [11], but more frequent than in Norway (25–37%) [37,51]. According to Zahn et al. [52], urban dwellers eat out more often, compared with suburban and rural residents.

In our study, gender did not influence the frequency of using canteen services. In previous studies, men used food services more often than women [11,37,38,51,52]. Moreover, skipping lunch was more common among men and women who did not have a canteen available. It was found that a lack of time during the working day forced employees to skip lunches or eat ready-to-eat meals [53].

These differences were also due to consumer education and financial status, especially high income. In our research, 81.7% of respondents had higher education and 78% had a very good or good financial situation. As indicated by other authors, a higher level of education and income [11,37,51,52,54,55,56] and work in locations with staff canteen located [54,55] are positively connected with the frequent use of staff canteens or restaurants. The staff restaurant/canteen is an example of the benefits that the employer can offer to employees to increase their ability to eat healthy meals, which are balanced according to dietary guidelines.

In our study, we made a thorough analysis (multidimensional calculations) of the reasons for not using the canteen and established specific consumer profiles related to these causes. Getting to know them has great practical implications and may contribute to increasing the use of canteens and even improving the eating habits of Poles. Our research shows that the main reason for not using staff restaurants was frequent dietary mistakes, i.e., snacking. It is worth emphasizing that financial status influences employee behavior regarding the use of canteens. For example, people who declared good financial status indicated different solutions in the area of food during their work (food delivery, fast food, other restaurants, or snacking). However, people whose financial status were “not good, not bad” preferred to eat at home. This is valuable information for canteen owners to extend the offer to employees of various financial status.

### 4.2. Factors Influencing the Use of Staff Canteens

In our research, the main factors influencing the use of staff canteens were location, price (including promotions and discounts), service quality, and consumer habits. Other factors (meetings, pleasant atmosphere, speed of service) were less important for the respondents. These results are similar to the results presented by other authors who also indicated location [56], price, or rather cost that reflects good value for money [17,39,53,57], and the quality of service [17,18,19,20,58,59]. Our results were in contrast to others in terms of portion size. The portion sizes of the foods eaten outside the home increased over the years [60]. For our respondents, the size of the portion is a very important element in the functioning of a canteen. This is probably due to increased nutrition knowledge and consumer awareness. Respondents preferred different sizes of meals, depending on their preferences; however, in staff canteens, there were no choices in portion size.

It seems to be that food in staff canteens is the most important for consumers. Based on the research result, the expectation of consumers in staff canteens is different from customers of a full-service restaurant, especially for upscale restaurants, which clients usually use for social contact [61]. Customers use canteens to eat meals; thus, food is important to them. In an earlier study [62], nutrition recommendation was important for consumers in everyday diets, but not important in a restaurant. For the respondents of staff canteens, quality and variety of meals and a pro-healthy menu (low calorie, vegetarian, fat, and cholesterol reduced, sodium content reduced, simple sugar content reduced, gluten-free, lactose-free) were important. The least important factors were special dishes, international cuisine, relax/rest, or formulating a meal yourself. Our research confirmed the results of other authors [63,64,65] that city dwellers, like our respondents, are interested in a healthy diet that requires adequate knowledge about food and nutrition. They more often choose organic products, and products cooked using healthy cooking methods [66,67,68,69]. Earlier studies [45,62] in Polish catering establishments showed that the sensory quality of food was the most important, while the nutritional value of meals in most establishments was not a priority. Dietary, easily digestible, low-calorie, light, fat-reduced, low-salt, and sugar-reduced meals, among others, being consistent with global trends were offered by only a few establishments, despite the positive attitude of consumers and catering staff toward them. However, in the last decade, customers started showing interest in rational nutrition recommended by nutritionists, especially those who use canteens and lunch restaurants.

In our study, the respondents expected a pro-healthy menu in staff canteens. Employees with higher education, as in our research, are more aware of health issues according to other authors [54,55]. As pointed out by other authors [37], healthy menus are often available at larger workplaces and those where the majority (>75%) of employees are women. Moreover, financial support from the company is positively associated with a healthy meal option. To attract customers, catering companies introduce all kinds of innovations such as healthy eating programs, sustainable development principles in all aspects of the organization, supporting Fairtrade, and philanthropy [46,47,70]. Workplace strategies, such as reducing barriers to healthy eating, can help employees follow healthy diet guidelines [37] and improve their diet quality. Researchers [71] suggested that price discounts or a voucher system could be an incentive for the consumption of healthier foods. The authors found that there is a tendency among young people to eat healthily and to consider the origin and composition of food and food waste [53], as well as life with the “sustainability” trend [65,72]. In particular, among young adults and women, as well as in social groups with a high level of education, a change in dietary behavior toward a vegetarian or vegan diet is observed [72].

Some of our respondents wanted traditional meals containing fats, sugar, and salt. This is similar to the results of other authors [73]. Consumers claimed that they would like healthier choices at restaurants, but their order was not “healthy”. Nutritional knowledge and attitudes to food are gender-specific around the world. Not only do women have a better knowledge of nutrition, but they also have a more positive attitude toward healthy eating than men [73,74,75].

### 4.3. Evaluation of Staff Canteens by Respondents

Identifying the reasons for customer dissatisfaction is a method of assessing consumer satisfaction. The calculations made by means of multidimensional cluster analysis showed high consumer satisfaction and a positive situation concerning small comments by employees on the operation of staff. In fact, they had very few remarks in all examined aspects. Hygiene in the areas visible to employees (tables, consumer room, meal delivery, etc.) was evaluated very positively. This may indicate the implementation of the obligatory hygiene requirements in food production in the canteens [76]. The atmosphere inside the canteen is directly related to the way the customer perceives a restaurant. The perception can be positive or negative considering the atmosphere and, therefore, can shape the overall level of customer satisfaction.

Understanding consumer expectations will help managers to meet them. The use of PCA allowed for the analysis of expectations in the context of consumer metrics. The respondents would like an increase in the variety of meals, including vegetarian and vegan dishes, salads, cooked vegetables, eggs, fish and poultry dishes, fruit, and fresh fruit juices in the menu of staff canteens. They also proposed reducing the number of pork and flour dishes, characteristic of Polish cuisine, in favor of pro-healthy, low-fat, light dishes. Thus, the trend characteristic of other countries is visible in the behavior of Polish consumers. The analysis of consumer expectations can be an inspiration for canteen managers to perform similar activities in order to enhance what they offer and increase customer satisfaction. Eating at work is often associated with increased energy and fat intake and decreased consumption of fruits and vegetables [38,60]. Canteen operators are often criticized for being nutrient-poor and energy-dense [77], and for promoting high-calorie foods that provide a high profit margin [78]. Lower status and economically disadvantaged people are less likely to report healthy food habits [37,51,79,80] or the purchase of food that complies with dietary guidelines. In particular, disadvantaged people consume fewer fruits and vegetables compared to higher socioeconomic groups [81,82,83].

The positive association between increased consumption of food prepared outside the home and the increasing prevalence of obesity is described as a major social challenge for health and well-being [57,84]. As indicated by authors [85], the choice of food served in staff canteens that is high in saturated fat, salt, and sugar is suitable based on work stress.

The selection of the sample was a limitation of the obtained test results. Nutritional behaviors in employee canteens were assessed, and consumer opinions of only a capital city were obtained, which may not translate into opinions from smaller cities and villages. The results relate rather to people with good financial status, while people with lower financial status may have a different opinion.

## 5. Conclusions

To assess consumer attitudes and behavior on the operation of staff canteens, including their expectations and comments, multidimensional statistical calculations were successfully used. Among consumers of staff canteens, four groups were distinguished, because of their expectations; “choice-oriented consumers” and “quality-oriented consumers” were the main groups, followed by “cost-oriented consumers” and “satisfied consumers” of staff canteens. It was revealed that the surveyed customers mostly valued the quality and variety of meals, as well as the portion size. Canteen customers follow global megatrends regarding “healthy” diets and a sustainable approach, and they expect changes toward the offer of healthy meals. They pointed out the need for an increase in the variety of meals, including the availability of vegetarian and vegan dishes, as well as health-promoting dishes. At the same time, customers make typical mistakes in nutrition by refusing to eat meals offered by canteens. The results provide the basis for practical implications for owners or managers of staff canteens, as they analyzed the needs and expectations of their potential customers. The results allowed us to analyze the current situation with regard to staff nutrition toward healthy living and to define assumptions for public health.

Our research results are practical. In employee canteens, the menu offered for employees of different financial status is important, as well as the choice of different meal sizes. This can be a reason to improve dietary behavior, with customers giving up on snacks and overeating in the future.

## Figures and Tables

**Table 1:** Characteristics of respondents.

Population Features	Group	Number of Respondents	Percentage of Respondents
Total	-	600	100.00
Gender	Female	270	45.00
	Male	330	55.00
Age	18–30 years old	424	70.67
	31–50 years old	176	29.33
Education	Secondary school	110	18.33
	Higher education (university)	490	81.67
Workplace	Office employee	378	63.00
	Management staff	222	37.00
Length of work in current place	Less than 2 years	248	41.33
	2–5 years	184	30.67
	6–10 years	92	15.33
	11–15 years	63	10.50
	Over 15 years	13	2.17
Financial status in own opinion	Very good	112	18.67
	Good	357	59.50
	Not good, not bad	131	21.83
	Bad	0	0.00

**Table 2:** Type of meals and frequency of eating at staff canteen by respondents (*n* = 600).

Type of Meals	Number of Answers	Average ± SD *	Age	*p*-Value	Workplace	Education
				Financial Status		
Cold breakfast	88	0.33 ± 0.99	NS	0.0036	NS	NS
Hot breakfast	88	0.33 ± 0.99	NS	0.0363	NS	NS
Soups	100	0.28 ± 0.78	NS	0.0004	NS	0.0078
Main course	381	1.41 ± 1.53	NS	0.0321	NS	0.0384
Vegetarian meals	554	2.86 ± 1.62	0.0018	NS	NS	0.0041
Fast food	221	0.56 ± 0.92	0.0012	0.0175	NS	NS
Cakes/pie	102	0.22 ± 0.58	0.0479	<0.0001	<0.0001	NS
Sandwiches	133	0.33 ± 0.79	0.0043	<0.0001	NS	0.0043
Salads	181	0.62 ± 1.22	NS	0.0006	NS	NS
Desserts	203	0.67 ± 1.18	NS	NS	NS	NS
Fruits	140	0.35 ± 0.81	NS	<0.0001	NS	NS
Beverages	81	0.25 ± 0.85	NS	NS	NS	0.0299

* Frequency of consumption of meals: (5)—every day, (4)—four times a week, (3)—three times a week, (2)—twice a week, (1)—once or less than once a week; (0)—never; SD—standard deviation, NS—not significant.

**Table 3:** Important elements of staff canteens indicated by respondents (*n* = 600).

Important Elements in Canteen Operation	Average ± SD	Q25	Median	Q75
Quality of meals	1.22 ± 0.47	1	1	1
Size of meals	1.22 ± 0.47	1	1	1
Variety of meals	1.84 ± 0.77	1	2	2
Exquisite meals	1.61 ± 0.62	1	2	2
Relaxing in a pleasant environment	2.22 ± 0.90	2	2	3
Quality of customer service	2.09 ± 0.87	2	2	3
Low prices of meals	1.67 ± 0.65	1	2	2
Promotion *	1.88 ± 0.86	1	2	2
Discounts **	2.22 ± 1.02	1	2	3
Possibility of ordering a meal with delivery	1.85 ± 0.96	1	2	2
Possibility of composing a meal on their own	2.61 ± 1.13	2	3	3
Traditional cuisine	2.19 ± 0.92	2	2	3
Possibility of take-away	2.16 ± 0.92	2	2	3
International cuisine	2.40 ± 1.04	2	3	3
Special dishes (chef dishes)	2.28 ± 0.94	2	2	3

Scale: (1)—very important, (2)—important, (3)—not important, (4)—completely unimportant; SD—standard deviation; * daily offer, seventh free coffee; ** discounts for companies, regular customers, passes.

**Table 4:** Evaluation of staff canteens by consumers.

Evaluation of Restaurant		Average ± SD	Q25	Median	Q75
Assessment of material factors in the canteen	Location	4.52 ±0.84	4	5	5
	Opening hours	4.26 ± 0.88	4	4	5
	Interior design	3.61 ± 0.97	3	4	4
	Atmosphere of the canteen	3.71 ± 1.03	3	4	4
	Cleanliness in the canteen	3.97 ± 0.86	4	4	4
	Availability of dishes and cutlery	3.97 ± 1.05	4	4	5
	Cleanliness of dishes and cutlery	3.82 ± 1.13	3	4	5
	Availability of sugar, spices, and napkins	3.76 ± 1.04	3	4	4
	Availability of trolleys for trays (to return the trays)	3.98 ± 1.02	4	4	5
	Availability and proximity of toilets	3.33 ± 1.38	3	4	4
Evaluation of customer service:	The way of welcoming clients	3.94 ± 1.08	4	4	5
	Speed of service	3.94 ± 1.08	4	4	5
	The queues for meals	4.06 ± 1.01	4	4	5
	Staff professionalism	3.61 ± 1.04	3	4	4
	Staff politeness	3.98 ± 0.96	4	4	5
	Staff outfit	4.05 ± 1.00	4	4	5
	Knowledge about meals service	4.01 ± 0.97	4	4	5
	Staff commitment to customer service	4.06 ± 1.01	4	4	5
Evaluation of menu:	Attractiveness of the menu	3.55 ± 0.96	3	4	4
	Availability of the menu	3.55 ± 0.96	3	4	4
	Taste of meals	3.65 ± 1.01	3	4	4
	Temperature of meals	3.55 ± 0.99	3	4	4
	Presentation of meals	3.44 ± 1.00	3	4	4
	Size of portions	3.69 ± 0.94	3	4	4
	Quality of soups (taste, smell, method of serving)	3.63 ± 0.92	3	4	4
	Quality of the main course (taste, smell, texture, etc.)	3.27 ± 1.42	3	4	4
	Variety of breakfast offer	3.53 ± 1.06	3	4	4
	Variety of salads offer	2.17 ± 1.90	0	3	4
	Variety of meals on the daily menu	2.53 ± 1.84	0	3	4
	Diversity of meals over a longer period of time	3.39 ± 1.17	3	4	4
	Pro-healthy culinary techniques	3.34 ± 1.20	3	4	4
	The possibility of buying commercial products	3.00 ± 2.22	2	3	4
	Prices of soups	3.62 ± 1.41	3	4	4
	Prices of main dishes	3.18 ± 1.35	3	4	4
	Prices in relation to quality	3.20 ± 1.10	3	3	4
	Readability of the menu, visibility of prices	3.25 ± 1.12	3	3	4
	Promotional actions *	3.53 ± 1.16	3	4	4
	Information about current discounts and promotions	2.41 ± 1.47	1	3	4
	Possibility to take away	2.37 ± 1.45	1	2	3
	Other additional services **	3.09 ± 1.71	3	4	4

Scale: 5—very good, 4—good, 3—average, 2—somewhat unsatisfactory, 1—poor; SD—standard deviation; * promotional actions (discounts: temporary, for regular customers, passes); ** other additional services (organization of name-day party for colleagues, very important person (VIP) services, etc.).

## Supplementary Materials

The following are available online at https://www.mdpi.com/1660-4601/17/17/6239/s1, Table S1: Questionnaire structure.
