# Supplementary material for: Attitudes and Consumer Behavior toward Foods Offered in Staff Canteens
Source: Int J Environ Res Public Health. 2020 Aug 27;17(17):6239. doi: 10.3390/ijerph17176239 (PMC7504587; doi:10.3390/ijerph17176239)
Supplement: Supplementary file 1 [file ijerph-17-06239-s001.pdf]

Table S1. Questionnaire structure.

Question	Variants of Answers
Frequency of staff canteen visits	(1) every day, (2) 4 times a week, (3) 3 times a week, (4) twice a week, (5) once or less than once a week
Factors influencing the use of staff canteen (multiple choice question)	quality, price, habits, location, possibility of eating in a pleasant atmosphere, meetings, others
Lunchtime at the staff canteen	at the beginning of the lunch break, in the middle of the lunch break, at the end of the lunch break, I have no set time
Time usually spent eating in a staff canteen	less than 15 minutes, 15–30 minutes, 31–45 minutes, 46–60 minutes, above 60 minutes
Frequency of dish and beverages consumption in staff canteens: cold breakfast, hot breakfast, soups, main course, vegetarian meals, fast food, cakes/pie, sandwiches, salads, desserts, fruits, beverages	(5) every day, (4) 4 times a week, (3) 3 times a week, (2) twice a week, (1) once or less than once a week, (0) never
Assessment of material factors in the staff canteens: location, opening hours, interior design, atmosphere of restaurant, cleanliness in the restaurant, availability of dishes and cutlery, cleanliness of dishes and cutlery, availability of sugar, spices and napkins, availability of trolleys for trays (to return the trays), availability and proximity of toilets	Scale: (5)—very good, (4)—good, (3)—average, (2)—somewhat unsatisfactory; (1)—poor
Evaluation of customer service: the way of welcoming clients, speed of service, queues for meals, staff professionalism, staff politeness, staff outfit, knowledge about meal service, staff commitment to customer service	Scale: (5)—very good, (4)—good, (3)—average, (2)—somewhat unsatisfactory; (1)—poor
Evaluation of menu: attractiveness, availability of the menu, taste of meals, temperature of meals, presentation of meals, size of portions, quality of soups (taste, smell, method of serving), quality of the main course (taste, smell, texture, the method of serving), variety of breakfast offer, variety of salads offer, variety of meals on the daily menu, diversity of meals over a longer period of time, pro-healthy culinary techniques, the possibility of buying commercial products (bars, drinks, others), prices of soups, prices of main dishes, prices in relation to quality, readability of the menu, visibility of prices, promotional actions (discounts: temporary, for regular customers, passes), availability of information about current discounts and promotions, possibility to take away, other additional services (organization of name-day party for colleagues, VIP services etc.)	Scale: (5)—very good, (4)—good, (3)—average, (2)—somewhat unsatisfactory; (1)—poor
Recommendation of staff canteen to other people	definitely yes, rather yes, neither yes or no, rather no, definitely no
What pro-healthy menu would you like to buy in staff canteens: low-calorie dishes, vegetarian meals, fat-reduced meals, cholesterol-reduced meals, sodium-reduced meals, simple carbohydrate-reduced meals, meals enriched with vitamins and minerals, high-fiber meals, gluten-free meals, lactose-free meals and others	choose the answer that suits you most (multiple choice question)
How important are the following elements in staff canteens: quality of meals, size of meals, variety of meals, exquisite meals, relaxing in a pleasant environment, quality of customer service, low prices of meals, promotion (daily offer, seventh free coffee), discounts, possibility of ordering a meal with delivery to the office, possibility of composing a meal on your own, traditional	Scale: (1)—very important, (2)—important, (3)—unimportant, (4)—completely unimportant

cuisine, possibility of take-away, international cuisine, special dishes (chef dishes)	
Reasons for rare use of the staff canteens: leaving meals during the day (skipping a meal), using the fast food offer outside the restaurant (fast food preferences), using other restaurants, snacks between meals (snacking), eating homemade food, delivering meals outside the office/ staff restaurant (food delivery), buying in a cafe or fast food bar (cafeteria preferences), eating at home	choose the answer that suits you most (multiple choice question)
Sociodemographic Data:	
Gender	women, men
Age	18–30 years old, 31–50 years old
Education	Secondary school, higher education (university)
Workplace	office employee, management staff
Assessment of financial status	very good, good, not good not bad, bad
Length of work in current place	less than 2 years, 2–5 years, 6–10 years, 11–15 years, over 15 years
